# Enhancing physical function and activity level in malnourished older adults through oral nutrition supplements: a randomized controlled trial

**DOI:** 10.1186/s12877-024-05164-1

**Published:** 2024-06-28

**Authors:** Ranil Jayawardena, Kalani Weerasinghe, Manoja Gamage, Andrew P. Hills

**Affiliations:** 1 Department of Physiology, Faculty of Medicine, University of Colombo, Colombo, Sri Lanka; 2 School of Exercise & Nutrition Sciences, Queensland University of Technology, Brisbane, Australia; 3 Health and Wellness Unit, Faculty of Medicine, University of Colombo, Colombo, Sri Lanka; 4 School of Health Sciences, College of Health and Medicine, University of Tasmania, Tasmania, Australia

**Keywords:** Oral nutritional supplement, Nutrition, Physical activity, Functional level, ONS, Older adults

## Abstract

**Background:**

Malnutrition of older individuals, leads to significant functional decline, reducing their quality of life. Lifestyle interventions; dietary improvements and supplementation are explored to enhance the physical function of older adults. The current study aimed to assess the impact of oral nutritional supplements (ONS) on the functional and activity levels of Sri Lankan older adults.

**Methods:**

This randomized controlled trial included; an intervention group (IG) receiving 200 mL of ONS providing 247 kcal per serving, for 12 weeks and a control group (CG) receiving an equivalent volume of water. Changes in handgrip strength, knee extension strength, gait speed, functional and activity levels were assessed.

**Results:**

The IG showed significant improvements in handgrip strength (43.96 ± 18.61 kg vs. 32.81 ± 17.92 kg; *p* < 0.001) and knee extension strength (23.45 ± 2.29 kg vs. 16.41 ± 2.09 kg; *p* < 0.001) following 12 weeks compared to the CG. The IG also exhibited significant improvements in gait speed (1.31 ± 0.52 m/s vs. 0.87 ± 0.26 m/s), Barthel index score, (0.30 ± 0.47 vs. -0.18 ± 0.66), PASE score (0.52 ± 17.79 vs. -1.60 ± 21.77) and IPAQ categories.

**Conclusions:**

ONS was found to be effective in improving the functional and physical activity levels of malnourished older adults.

**Trial registration.:**

Sri Lanka Clinical Trial Registry SLCTR/2022/021. Registered on 06/10/2022.

## Background

An increase in life expectancy leads to consistent growth in the older adult population [1]. The number of adults aged 60 years and above is projected to rise to approximately 20% by 2050 worldwide, up from 12.3% in 2015 [2]. In Sri Lanka, the prevalence of malnutrition, risk of malnutrition, and well-nutrition among community-dwelling older individuals have been estimated as 12.5%, 52.4%, and 35.1%, respectively [3]. These results indicate that nearly two-thirds of older adults are either experiencing malnutrition or are at risk of malnutrition [3]. A study by Hai and colleagues in Singapore provided evidence that low, mild-moderate, and severe malnutrition were associated with a higher risk of physical disability [4]. Severe malnutrition risk was also linked to significant functional decline in older individuals without disability [4]. Malnutrition among older adults is a critically important challenge, as it significantly burdens psycho-emotional and physical well-being, ultimately leading to a decline in overall quality of life (QOL) [5].

Ageing is a multifaceted process characterized by interconnected molecular, cellular, physiological, and functional changes [6]. Evidence points to the commencement of a linear decline in skeletal muscle mass and strength from as early as the fourth decade of life. By the eighth decade of life, up to 50% of muscle mass may be lost (sarcopenia), leading to a loss of function, disability, and frailty among many older adults. Declines in physiological and functional capacity lead to the onset of chronic inflammation and disruptions in energy metabolism, including variations in insulin sensitivity. These changes further influence neuronal and sensory functions, particularly vision, hearing, and mobility [7]. The musculoskeletal system is significantly affected by biological aging, resulting in the loss of muscle mass and bone density. This decline in musculoskeletal health contributes to increased physical frailty, susceptibility to falls, fractures, and loss of independence [8]. Additionally, reduced appetite, chewing ability, weight loss, fatigue, weakness, slow walking speed, and physical inactivity are often associated with ageing, ultimately leading to frailty and a drastic drop in QOL [9]. Furthermore, in Sri Lanka, the older adult population often experiences these symptoms without adequate dietary support, exacerbating malnutrition and diminishing QOL, particularly as traditional social support structures erode in many communities, such as the decline in multigenerational households [3].

The perception of distance to food outlets can also influence food insecurity among older adults with disabilities, contributing to malnutrition [10]. Malnutrition, in turn, leads to reductions in muscle mass and strength, resulting in decreased physical function, and creating a vicious cycle [10]. Improving diet quality, implementing supplementation, and incorporating appropriate physical activity and exercise are important lifestyle interventions to enhance the physical function of older adults [11]. A recent randomized controlled trial (RCT) reported that an acute dietary intake of nitrate resulted in a significant increase of 10.9% in maximal velocity of knee extension and a 4.4% increase (*p* < 0.05) in maximal knee extensor power among older individuals. The study results confirmed that this supplementation is efficacious in older adults, as diminished muscle function in this population can contribute to functional restrictions, dependence, and even premature mortality [12]. In another trial, the group receiving whey protein isolate along with bio-actives (± polyphenols and omega-3 fish oil) showed a significant improvement of 13% in knee extension strength (KES) compared to the group receiving carbohydrates and placebo capsules [13]. The whey protein isolate group also demonstrated the greatest improvement in gait speed, an 8% increase [13]. Another study conducted to investigate the effectiveness of creatine supplementation on strength and fat-free mass (FFM) development in older adults confirmed that resistance training can safely improve muscle strength and functional capacity in this demographic [14]. Moreover, the inclusion of creatine in the exercise regimen facilitated additional increases in both total mass and FFM, along with improvements in indices of isometric muscle strength [14]. Matheson and his research team conducted a trial to investigate the impact of specialized oral nutritional supplements (ONS) on hand grip strength (HGS) and its correlation with nutritional status in older adults with malnutrition [15]. Throughout their hospital stay, and up to 90 days after discharge, participants were administered standard care along with a high-protein and beta-hydroxy-beta-methyl butyrate (HMB) containing ONS or a placebo supplement (n = 324), with the aim of consuming two servings per day. HGS measurements were assessed using a dynamometer at baseline, hospital discharge, day 30, 60, and 90 post-discharge. The supplementation regimen effectively improved HGS in malnourished older adults, suggesting its potential contribution to overall patient recovery.

In this context, we have designed the present RCT to investigate the effectiveness of ONS in improving the functional status and physical activity levels of institutionalized, malnourished older adults in Sri Lanka. This study addresses a critical gap in the existing research by focusing on a population that is both under-studied and vulnerable. Hence, we hypothesize that ONS will positively impact physical function, which includes key elements such as KES, HGS, and overall mobility.

## Methods

The CONSORT 2010 statement guidelines regarding clinical trials (www.consort-statement.org) were followed for this RCT [16].

### Trial design and study setting

**Fig. 1:**
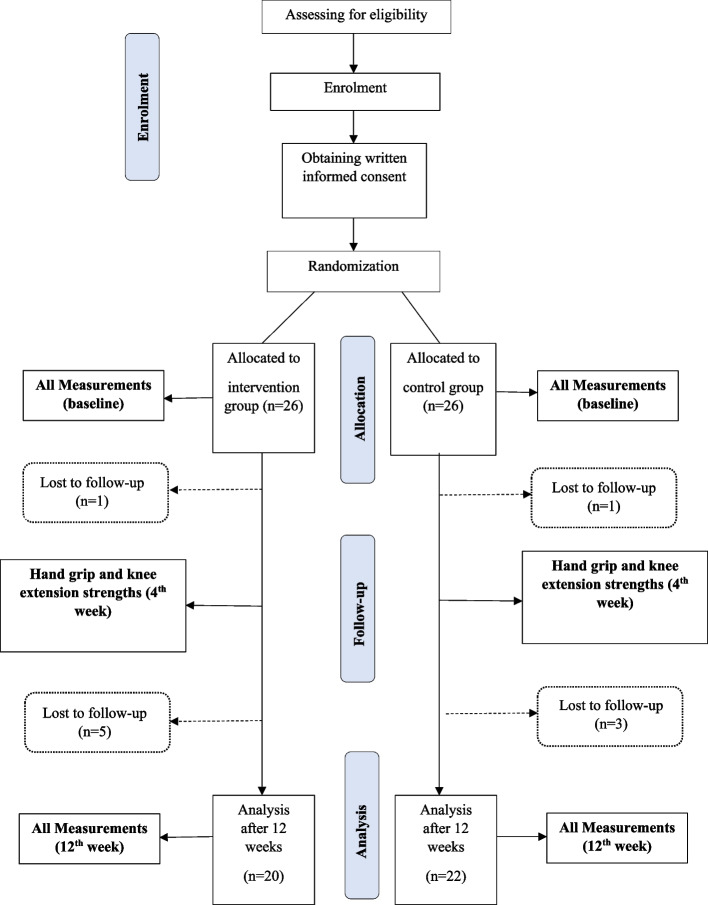
Flow diagram of enrolment, allocation, follow-up and analysis

The study was a single-centre, open-label, parallel-group, RCT within a designated residential care facility registered with the National Secretariat for elders in Colombo, Sri Lanka. Two parallel study groups were established, including an intervention group (IG) and a control group (CG), both within the same study setting. The study commenced with a screening visit to assess and confirm the eligibility of participants. Figure 1 describes the CONSORT flow diagram of this RCT.

### The ethical approval

Informed written consent was obtained from each participant, ensuring their voluntary participation and understanding of the study procedures and potential risks involved. The study procedures were conducted in accordance with the principles outlined in the Helsinki Declaration, ensuring the ethical treatment of participants and safeguarding their rights and well-being [17]. The study received ethical approval from the Sri Lanka Medical Association (ERC/22–005). The trial was registered at the Sri Lanka Clinical Trials Registry (SLCTR/2022/021) to ensure transparency and compliance with regulatory requirements.

### Sample size

The sample size calculation was derived from prior studies suggesting that an ONS intervention could result in a clinically significant weight gain of 5% among older adults, as indicated for improving nutritional status [18]. Consequently, a total of 50 eligible older adults were recruited and randomly assigned to either the IG (n = 25) or the CG (n = 25). The details of the sample size calculation are mentioned in the protocol paper, which was published in an open-access journal [19].

### Recruitment

Older adults (age ≥ 60) residing in a selected institution for a minimum of one year were screened using the MNA to assess their nutritional status [20]. Based on the MNA guidelines, individuals can be categorized into three distinct nutritional status levels: well-nourished (scoring 12–14 points), at risk for malnutrition (scoring 8–11 points), and undernourished (scoring less than 8 points) [21]. Those who obtained an MNA score equal to or less than 11 and met all other eligibility criteria mentioned earlier were recruited for the trial. This specific cut-off value was chosen to identify individuals who may benefit from nutritional intervention, in accordance with the tool's established guidelines and recommendations.

### Randomization and blinding

Eligible participants were randomized equally into IG and CG using a simple random sampling technique with an allocation ratio of 1:1. The randomization assignment was generated by an independent investigator using an online random number generation program. To ensure concealment of the allocation, sealed opaque envelopes were prepared in advance, indicating the allocation for each participant based on their recruitment number.

### Intervention

A glass of 200 mL of the ONS was administered for a duration of 12 weeks as the intervention. It was prepared by adding 4 level scoops (57 g) of Entrasol Platinum (containing 12 g of protein and 247 kcal of energy) from Kalbe Pvt. Ltd. to 200 mL of lukewarm water in addition to their usual diet. The nutritional composition of the ONS and more details about the intervention have been published elsewhere [19]. The ONS was administered by caretakers in the elderly care home, under the supervision of the research team, and was monitored daily through online video calls (WhatsApp). Thus, it can be said that this study was conducted in a controlled environment, ensuring zero contamination and 100% compliance. Participants assigned to the IG consumed the ONS before bed on a daily basis for 12 weeks, whereas participants in the control arm were placed on a waitlist and received a glass of water.

### Outcomes

The demographic details and anthropometry of each participant were collected. Differences in mean changes of variables including KES and HGS (both on the dominant side in kg and each measurement was taken three times), were assessed at baseline and the end of the 4^th^ and 12^th^ week of the intervention.

The peak force of the dominant leg’s KES was measured using a Lafayette Manual Muscle Tester (Model 01163, Lafayette Instrument Company, Lafayette, IN, USA). HGS was measured using a hydraulic handheld dynamometer (Model SH5001; SAEHAN Corporation, YangdeokDong, Masan, South Korea). For men, an HGS of less than 27 kg is considered indicative of the risk of malnutrition and muscle wasting, while for women, the threshold is less than 16 kg, according to the EWGSOP2 sarcopenia cut-off points [22].

Gait speed was measured by averaging two readings in the 6 m gait speed test. Activities in daily living (ADLs) and physical activity levels were collected between both the IG and CG at baseline at the end of the 12^th^ week.

The ability to perform ADLs was assessed using the Barthel index, a widely recognized tool for measuring an individual's level of functional status [23]. It evaluates the ability to perform basic self-care tasks such as feeding, grooming, bathing, and mobility. A higher score indicates better function. Each item is rated based on whether the patient can perform the task independently, with some assistance, or is dependent on help, as observed (0 = unable, 1 = needs help, 2 = independent) [24]. Scores between 24 and 30 suggest a healthy state, but scores between 17 and 23.5 indicate a risk of malnutrition. Scores lower than 17 indicate malnutrition [25].

The physical activity level was assessed using data collection tools, namely the PASE and IPAQ. The PASE questionnaire is a validated self-administered tool used for evaluating the physical activity of older adults [26]. It assesses a wide range of activities, including leisure, household, and occupational activities, providing insights into how their activity levels influence their overall health and well-being [27]. The IPAQ, on the other hand, is a self-report questionnaire designed to gauge an individual's physical activity levels and sedentary behaviour. It quantifies the frequency and duration of various types of physical activities [28]. The individuals can be classified as having high Metabolic Equivalent of Task (MET > 3000), moderate (MET 600–3000), or low physical activity (MET < 600) levels based on the MET scores obtained using the IPAQ [29].

The procedures followed in measuring each outcome are explained precisely elsewhere [19].

### Statistical methods

Data were analyzed using the Statistical Package for the Social Sciences (SPSS 20.0), and the significance level was set at 0.05, with a confidence interval of 95%. Descriptive statistics were employed to analyse the socio-demographic characteristics of participants. For categorical parameters such as gender and IPAQ score, Chi-Square tests were used to assess significance. Furthermore, an independent samples t-test was conducted to compare the efficacy of the ONS in enhancing the physical function parameters. Additionally, a dependent samples t-test was used to evaluate improvements in physical function parameters within both groups pre- and post-intervention.

## Results

Out of the 50 participants initially enrolled in the trial, 20 in the IG and 22 in the CG completed the 12-week study (Fig. 1). Results are reported for the 42 participants who completed the study using the per-protocol analysis method. The mean age of the IG was 75.38 ± 6.05 years, with a gender distribution of 8 males (32%) and 17 females (68%). The CG had a mean age of 74.84 ± 5.22 years, with 6 males (24%) and 19 females (76%). The baseline nutritional status of participants was assessed as being at risk of malnutrition with respect to their baseline MNA scores. Participants in the IG recorded scores of 8.72 ± 1.95, while those in the CG recorded scores of 9.56 ± 1.45 (*p*-value = 0.120). Table 1 displays the baseline characteristics of the study population, including demographic, anthropometric, and physical function parameter measurements. The baseline parameters indicate that 84.6% of male participants and 69.4% of female participants exhibited HGS below the established cut-off points, suggesting a significant risk of muscle wasting. At baseline, the Barthel Index (BI) scores were 19.15 ± 1.14 for the IG and 19.05 ± 1.69 for the CG (p = 0.740), indicating similar levels of independence in ADL between the groups.

**Table 1:** Baseline demographic, anthropometric, and physical function parameter measurements of study participants

Variable	IG (*n* = 20) Mean ± SD	CG (*n* = 22) Mean ± SD	*p* value
Age (years)	75.4 ± 6.1	74.8 ± 5.2	*p* = 0.730
Gender			*p* = 0.529
Male	7 (35%)	6 (27%)	
Female	13 (65%)	16 (73%)	
Duration of institutionalization (years)	5.20 ± 5.23	4.49 ± 4.22	*p* = 0.616
MNA score	8.72 ± 1.95	9.56 ± 1.45	*p* = 0.120
BMI (kgm^−2^)	18.98 ± 2.28	18.60 ± 2.24	*p* = 0.552
Dietary intake			
Energy intake (kcal)	1312.43 ± 186.08	1348.03 ± 207.84	*p* = 0.525
Carbohydrate intake (g)	208.30 ± 35.97	216.72 ± 37.02	*p* = 0.418
Fat intake (g)	37.00 ± 8.31	37.20 ± 8.68	*p* = 0.937
Protein intake (g)	34.55 ± 6.66	34.69 ± 6.47	*p* = 0.942
Gait speed (ms^−1^)	0.94 ± 0.35	0.97 ± 0.34	*p* = 0.967
BI score	19.15 ± 1.14	19.00 ± 1.69	*p* = 0.740
IPAQ score (number of subjects)			*p* = 1.000
Low active (< 600)	10 (50%)	10 (45.45%)	
Moderately active (600- 3000)	10 (50%)	12 (54.54%)	
PASE score	47.31 ± 30.02	45.69 ± 24.55	*p* = 0.849
KES (kg)	16.85 ± 2.34	16.28 ± 1.85	*p* = 0.878
HGS (kg)	37.06 ± 17.12	36.52 ± 15.39	*p* = 0.903
Male	22.03 ± 8.72	19.70 ± 3.60	
Female	14.07 ± 3.92	14.87 ± 5.84	

*BMI* Body Mass Index, *MNA* Mini Nutritional Assessment, *IPAQ* International Physical Activity Questionnaire, *IG* Intervention Group, *CG* Control Group, *PASE* Physical Activity Scale for the Elderly, *HGS* Hand Grip Strength, *KES* Knee Extension Strength

**Fig. 2:**
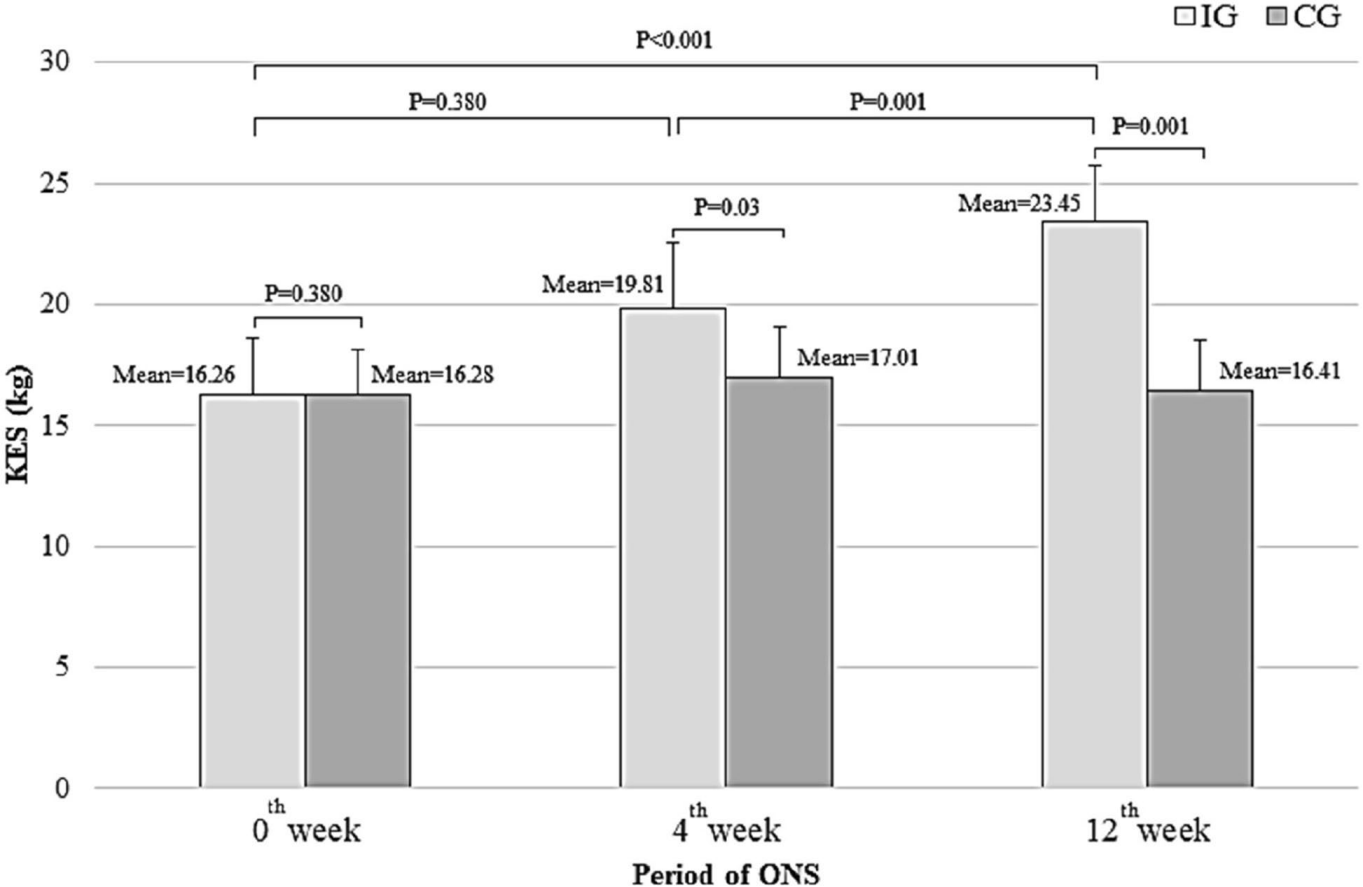
Comparison of the mean KES between IG and CG

**Fig. 3:**
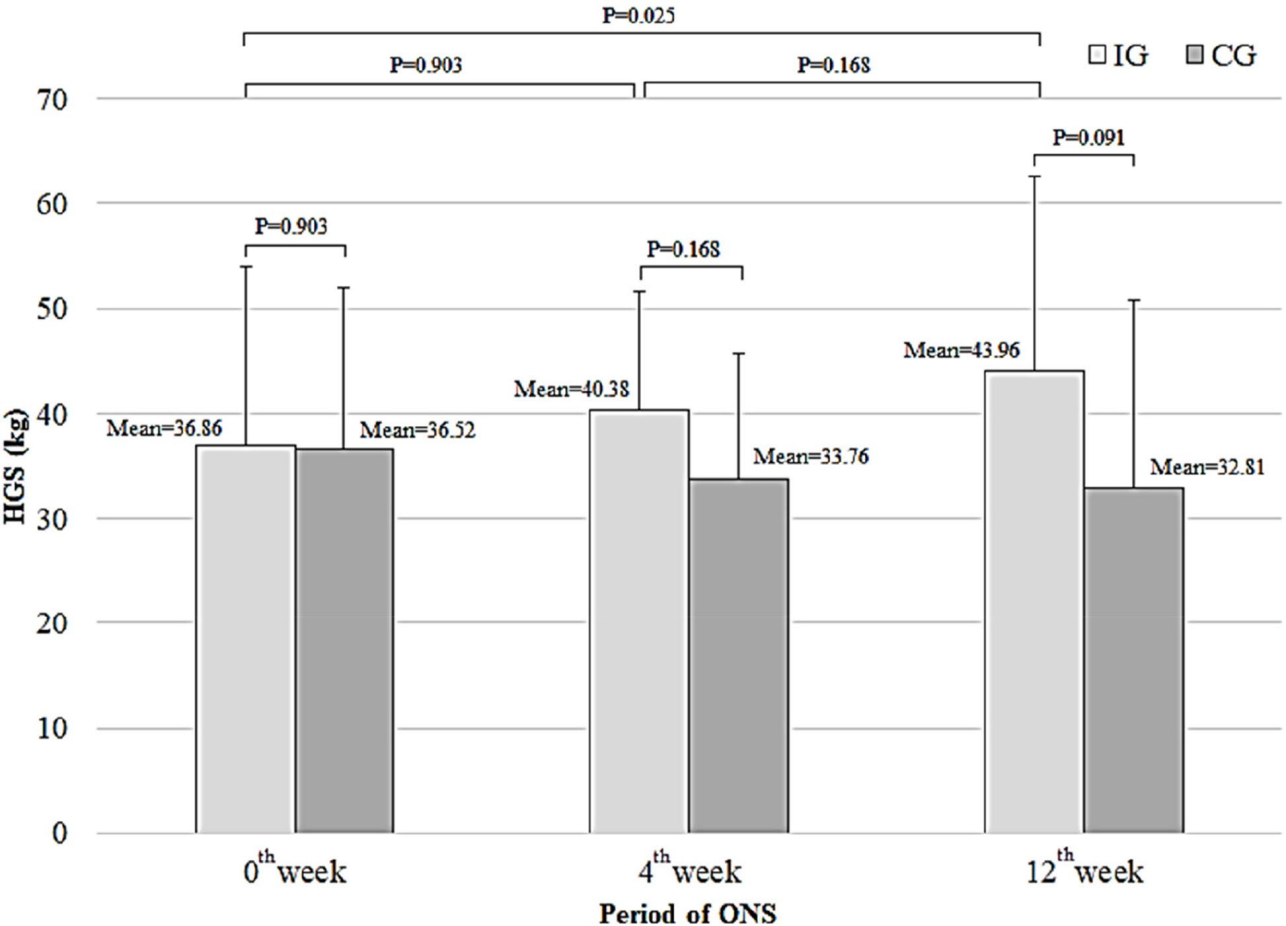
Comparison of the mean HGS between IG and CG

Following the ONS supplementation period, a significant improvement in the mean KES was observed in the 4^th^ week (19.81 ± 2.73 kg vs. 17.01 ± 2.07 kg;* p* = 0.03) and in the 12^th^ week (23.45 ± 2.29 kg vs. 16.41 ± 2.09 kg;* p* = 0.001) (Fig. 2). Similarly, a significant improvement in the mean HGS was observed at the end of the 12 weeks (43.96 ± 18.61 kg vs. 32.81 ± 17.92 kg;* p* = 0.002) among participants in the IG compared to those in the CG (Fig. 3). Furthermore, the changes in KES (*p* < 0.001) and HGS (*p* = 0.025) measurements were significantly enhanced following the ONS intervention in the IG compared to their corresponding baseline values. Conversely, these measurements decreased in the CG, as illustrated in Figs. 2 and 3.

At the end of the intervention period, participants in the IG exhibited significant improvements in gait speed (1.31 ± 0.52 m/s vs. 0.87 ± 0.26 m/s;* p* < 0.05), PASE score (0.52 ± 17.79 vs. -1.60 ± 21.77;* p* < 0.05), and IPAQ (*p* < 0.05) (Table 2). The proportion of participants classified as 'low active' was 5 (25%) in the IG and 13 (59.09%) in the CG, and those classified as 'moderately active', 15 (75%) in the IG and 9 (40.90%) in the CG following 12 weeks of oral nutritional supplementation. Furthermore, the BI scores improved to 19.45 ± 1.61 for the IG, whereas the same variable deteriorated to 18.87 ± 1.84 for the CG (p < 0.05). The change in BI scores over the 12 weeks was 0.30 ± 0.47 for the IG and -0.18 ± 0.66 for the CG (p < 0.05) (Table 2).

**Table 2:** Comparison of the changes in gait speed, BI, IPAQ, and PASE scores between the IG and the CG

Variable	IG (*n* = 20) Mean ± SD	CG (*n* = 22) Mean ± SD	*p* value
Gait speed (m/s)	1.31 ± 0.52	0.87 ± 0.26	*p* < 0.05
BI score	0.30 ± 0.47	-0.18 ± 0.66	*p* < 0.05
IPAQ score (number of subjects)			*p* < 0.05
Low active (< 600)	5 (25%)	13 (59.09%)	
Moderately active (600- 3000)	15 (75%)	9 (40.90%)	
PASE score	1.52 ± 17.79	-1.60 ± 21.77	*p* < 0.05

*BI* Barthel index, *IPAQ* International Physical Activity Questionnaire, *IG* Intervention Group, *CG* Control Group, *PASE* Physical Activity Scale for the Elderly, *HGS* Hand Grip Strength, *KES* Knee Extension Strength

## Discussion

The key findings of this 12-week ONS supplementation trial were that there were significant improvements in several physical functioning and activity parameters in the test group compared to the controls. These included HGS, KES, gait speed, and Barthel index scores, as well as physical activity levels measured by the PASE and the IPAQ. Interestingly, all outcome measures also exhibited a notable enhancement in the IG compared to their respective pre-intervention results. While a decline in muscle mass and strength is generally observed with aging, our study demonstrates that a significant number of participants were able to maintain or even enhance their muscle mass and strength, likely due to the effects of the ONS supplementation provided during the trial. This suggests that ONS can play a critical role in mitigating the typical musculoskeletal deterioration seen in older adults.

The increase in life expectancy worldwide has led to a growing older adult population, and the prevalence of malnutrition among older individuals is a global issue [1]. The proportion of people aged 60 or above was 9.8% in 2017 in South East Asia. It is predicted to increase to 13.7% and 20.3% by 2030 and 2050, respectively [1]. Sri Lanka is one of the South Asian countries with a rapidly ageing population, undergoing a rapid epidemiological and nutritional transition. With the improvement of healthcare facilities in Sri Lanka, the older adult population has been increasing gradually during the past few decades. It is estimated that one in four Sri Lankans will be an older adult person by the year 2041 making Sri Lankans the oldest population in South Asia [30]. It has also been reported that over 50% of elders in some older adult care institutions in Sri Lanka are malnourished [31]. The same research publication has stated that a considerably higher proportion (59.1%) of older adult people living in care homes in Kandy were at risk of being malnourished while 3.8% were malnourished [31]. According to another study conducted in a community-dwelling in the Kandy district, 12.5% of the adults were malnourished whereas about half (52.4%) were at risk of malnutrition [3]. A similar kind of community-based study in relation to nutritional status in the Galle district has stated that 0.5% of older adults were malnourished and 30.8% were at risk of malnutrition [32]. Malnutrition is prevalent among older adults living in care institutions due to factors such as inadequate staffing, poor individualized care, lack of nutritional monitoring, and potentially limited knowledge about nutritional needs [3].

Although regions across the world are experiencing similar demographic shifts, the prevalence of malnutrition in older adults varied considerably due to variations in both the tool used for its assessment and the respective population [33]. The prevalence, factors contributing to malnutrition and the strategies to address may be influenced by a multitude of factors across various geographic and socio-economic contexts, cultural practices and healthcare infrastructures. Meanwhile, our study was aimed to assess the impact of ONS in addressing malnutrition among older adults in Sri Lanka.

Weak grip strength, which is indicative of sarcopenia, has been consistently associated with poor health outcomes [34]. Our study findings are consistent with the significant improvements reported in HGS on ONS in previous literature. Previous research has indicated that a 12-week supplementation of a mixture of nutrients, including HMB, arginine, and lysine, resulted in a notable enhancement of HGS and positive trends in FFM [35]. However, our ONS did not contain HMB but did include 12 g of high-quality protein, including amino acids such as arginine and lysine. Therefore, we believe the findings of our study are based primarily on the adequate intake of energy and high-quality protein. Both findings indicate that this ONS positively impacts functionality, strength, FFM, and protein synthesis among older adults [35].

HGS is also an important marker of the health of the arm muscles, so important in ADLs [36] such as opening medication containers, carrying grocery bags, pushing doors, and using kitchen utensils. Many tasks become markedly more challenging when HGS is diminished and can lead to further restrictions in ADLs, including reducing the frequency one can leave their home and negatively impacting their functional, psychological, and social well-being [37]. An optimal hand grip strength is not only of great importance in activities related to feeding, and personal hygiene tasks, like bathing and grooming, but also in leisure-time hobbies. Recreational pursuits including playing a musical instrument, painting, knitting, or gardening, may be impacted by poor overall health as reflected by grip strength. This also extends to handling household tools like hammers, screwdrivers, or wrenches during simple repairs or maintenance around the house [37]. Hence, it is crucial to maintain HGS, as it is also related to joint mobility and muscle strength, and ultimately contributes to an improved QOL among the older adult population [38].

A significant finding was that participants who received ONS demonstrated the greatest improvement in KES. A study by Liao and colleagues investigated the efficacy of a combined intervention involving resistance exercise training and protein supplementation in promoting the recovery of walking speed among individuals with knee osteoarthritis and sarcopenia [39] and found that the 12-week treatment enhanced the therapeutic effects by accelerating the time required for walking speed recovery to a level equal to or greater than 1.0 m/s. This improvement in walking speed not only helps reduce the severity of the disease but also has the potential to minimize the risk of sarcopenia in these patients [39]. In another RCT, performing thirty minutes of heavy-load strength training three times per week, along with protein supplementation (34 g of milk protein each day, giving 149 kcal), resulted in increased leg lean mass, as well as improvements in strength and functional capacity among older adults with limited mobility [40].

Perhaps the most striking finding of the current study was the substantial increase in the physical activity level measured by the PASE and the IPAQ among the IG participants in comparison to the CG. This result can be attributed to an improvement in muscular strength due to the implementation of the ONS intervention. The enhancement of physical activity levels is directly linked to several critical clinical outcomes. Firstly, increased physical function can significantly decrease the risk of falls, which are a major health concern for older adults [41]. Improved muscle strength and balance from heightened physical activity can help maintain mobility and reduce the incidence of fall-related injuries, which often lead to long-term disability. Secondly, the capacity for independent living is closely associated with physical function. Higher levels of muscle strength and mobility allow older adults to perform daily activities more efficiently and without assistance [42]. This independence is not only crucial for improving QOL but also reduces the burden on caregivers and healthcare systems [43]. Moreover, enhanced physical function is linked to improved metabolic health [44]. Increased muscle mass can improve metabolic rate, helping to regulate blood sugar levels and reduce the incidence of metabolic diseases, which are prevalent in older populations. Lastly, the psychological benefits associated with improved physical health cannot be overlooked. Better physical health often leads to improved mental health, with reductions in symptoms of depression and anxiety, which are common among older adults [45]. This psychological improvement is likely due to increased social interaction and greater engagement in community and personal activities, facilitated by better physical health [46].

Similar outcomes were observed in a trial conducted by Yoshimura and team [47], wherein a group of older adults in a rehabilitation hospital underwent a combination of resistance training and ONS for a period of 2—6 months. Another parallel study examining the effects of a hyperproteic, hypercaloric ONS combined with a standardized physical intervention on the functional status and QOL of frail institutionalized older adults [48] also yielded promising results. Improvements were observed in the level of physical functionality measured using the Short Physical Performance Battery and the Short-Form Late-Life Function and Disability Instrument. Additionally, participants exhibited enhanced QOL, particularly among those with greater frailty criteria, lower functional levels, lower vitamin D levels, and poorer nutritional status. Researchers concluded that the 12-week intervention involving ONS alongside physical exercise not only improves function and muscle strength but also enhances their overall QOL.

This improvement in walking speed not only helps reduce the severity of the disease but also has the potential to minimize to risk of sarcopenia in these patients’ The key strength of the present study was the high rate of follow-up, with all participants demonstrating 100% compliance and contamination throughout the study due to the high level of supervision provided by the research team and institutional caretakers. Additionally, the assessment of physical function parameters at multiple time points, including baseline, the 4^th ^week and the 12^th^ week following supplementation, provided valuable insights into the effects of ONS over time. Another notable strength of the current study is that we observed improvements in the functional status and physical activity levels of malnourished older adults solely through ONS supplementation without incorporating any prescription of any exercise intervention or physical activity regimen. As a consequence, this straightforward nutritional intervention makes it easily replicable in any setting.

However, several limitations of the current study need to be acknowledged. The study was limited to a single centre with only enrolled institutionalized older adults, with a follow-up period of 12 weeks. This may limit the generalizability and applicability of our findings to a broader population. Moreover, the absence of blinding could introduce performance bias among participants, as they were aware that they were consuming ONS. Additionally, our study focused solely on the hand grip and muscle strength of the knee extensor muscles, which may not reflect the strength of all other muscle groups in the body. Furthermore, since we specifically recruited malnourished older adults, we were not able to predict the effects of ONS on older adult populations with normal nutritional status. The role of chronic illnesses and comorbidities on the effect of ONS was not specifically identified in our study.

Future trials should consider the effects of ONS on a larger sample of community-based older adults with varying nutritional statuses and extend the follow-up period to assess the long-term effects of ONS on physical activity. Studies identifying the role played by chronic illnesses in assessing the effects of ONS on older adult populations will also contribute to a more comprehensive understanding of the potential benefits and considerations in diverse health contexts. Additionally, it would be interesting to design a multimodal approach that combines nutritional supplementation with varying content of energy, protein, micronutrients, and nutraceuticals to assess functionality. Such research would assist in providing a more holistic understanding of the potential benefits of ONS in different older adult populations.

In our study, we aimed to investigate the effects of a 12-week ONS intervention on physical functioning parameters in malnourished older adults. While our primary focus was on addressing malnutrition, the underlying mechanisms regulating energy homeostasis and appetite may change as people age [49]. We recognize the importance of considering the broader context of energy homeostasis and appetite regulation in this population. They are multifaceted processes that are influenced by a variety of factors, including olfactory stimuli, stretch receptor signals originating in the stomach and proximal small intestine, nutrients such as glucose and amino acids, metabolites like lactate, pyruvate, and ketones, and alterations in gut hormones including cholecystokinin, glucagon-like peptide-1 and ghrelin (in response to nutrient ingestion) and the hypothalamus [49]. These factors play a crucial role in determining meal size, nutrient absorption, and overall nutritional status in older adults [49]. It is important to acknowledge that while our study demonstrated significant improvements in physical functioning parameters, including HGS and KES, as well as increased physical activity levels, we did not directly investigate the underlying mechanisms. Future research may benefit from exploring these mechanisms in greater detail, especially in the context of ageing and malnutrition, to provide a more comprehensive understanding of the factors influencing dietary behaviour and nutritional outcomes.

As future recommendations, it can be stated that the findings of this trial may influence healthcare practices in older adults living in care facilities. Regular nutritional assessments and personalized nutritional interventions are suggested as standard practices to combat malnutrition and its associated risks.

## Conclusion

In conclusion, the results of this clinical trial demonstrated that a 12-week supplementation with ONS, which provides nearly 250 kcal, leads to significant improvements in KES, HGS, and gait speed among malnourished older adults when compared to controls. Furthermore, these enhancements in muscle strength are likely associated with improvements in functional status, as exhibited by increases in Barthel Index scores, PASE, and IPAQ scores. Therefore, supplementation with ONS as a bedtime drink was found to be effective in improving the physical activity level and functional status of malnourished older adults.

## Data Availability

The datasets generated and/or analyzed during the current study are not publicly available. Due to the nature of the industrial collaboration, our partner prefers to withhold raw data to safeguard confidential information. However, the data are available from the corresponding author upon reasonable request.

## Abbreviations

ADLs: Activities in daily living
QOL: Quality of life
RCT: Randomized controlled trial
HGS: Hand grip strength
KES: Knee Extension Strength
ONS: Oral nutritional supplements
HMB: Beta-hydroxy-beta-methyl butyrate
FFM: Fat-free mass
PASE: Physical Activity Scale for the Elderly
IPAQ: International Physical Activity Questionnaire
IG: Intervention group
CG: Control group
MNA: Mini Nutritional Assessment
SPSS: Statistical Package for the Social Sciences
